# Transcriptome-based network analysis related to M2-like tumor-associated macrophage infiltration identified VARS1 as a potential target for improving melanoma immunotherapy efficacy

**DOI:** 10.1186/s12967-022-03686-z

**Published:** 2022-10-27

**Authors:** Zhengquan Wu, Ke Lei, Huaizhi Li, Jiali He, Enxian Shi

**Affiliations:** 1 Department of Otorhinolaryngology, Head and Neck Surgery, University of Munich, 81377 Munich, Germany; 2 Walter Brendel Center for Experimental Medicine, University of Munich, 81377 Munich, Germany; 3 Department of Dermatology, The Second People’s Hospital of Chengdu, 610021 Chengdu, People’s Republic of China; 4 Department of Endocrinology, Shenzhen University General Hospital, Shenzhen University, 518055 Shenzhen, People’s Republic of China; 5 Shenzhen Healthcare Committee Office, 518020 Shenzhen, People’s Republic of China

**Keywords:** Tumor associated macrophages, Melanoma, Macrophage polarization, Immunotherapy, Prognostic model, VARS1

## Abstract

**Rationale:**

The M2-like tumor-associated macrophages (TAMs) are independent prognostic factors in melanoma.

**Methods:**

We performed weighted gene co-expression network analysis (WGCNA) to identify the module most correlated with M2-like TAMs. The Cancer Genome Atlas (TCGA) patients were classified into two clusters that differed based on prognosis and biological function, with consensus clustering. A prognostic model was established based on the differentially expressed genes (DEGs) of the two clusters. We investigated the difference in immune cell infiltration and immune response-related gene expression between the high and low risk score groups.

**Results:**

The risk score was defined as an independent prognostic value in melanoma. *VARS1* was a hub gene in the M2-like macrophage-associated WGCNA module that the DepMap portal demonstrated was necessary for melanoma growth. Overexpressing *VARS1 in vitro* increased melanoma cell migration and invasion, while downregulating *VARS1* had the opposite result. *VARS1* overexpression promoted M2 macrophage polarization and increased TGF-β1 concentrations in tumor cell supernatant in vitro. VARS1 expression was inversely correlated with immune-related signaling pathways and the expression of several immune checkpoint genes. In addition, the VARS1 expression level helped predict the response to anti-PD-1 immunotherapy. Pan-cancer analysis demonstrated that *VARS1* expression negatively correlated with CD8 T cell infiltration and the immune response-related pathways in most cancers.

**Conclusion:**

We established an M2-like TAM-related prognostic model for melanoma and explored the role of VARS1 in melanoma progression, M2 macrophage polarization, and the development of immunotherapy resistance.

**Supplementary information:**

The online version contains supplementary material available at 10.1186/s12967-022-03686-z.

## Introduction

Melanoma is a highly aggressive skin cancer with early metastases and have the highest mortality rate in skin cancer [43]. Its incidence has increased in recent years and it has become one of the fastest growing tumors. Diagnosis rates are also increasing among young people [44]. Despite the recent advances in neoadjuvant immunotherapy, chemotherapy, and targeted therapy improving patient prognosis, many patients only achieve temporary remission and eventually develop therapy resistance. Therefore, the mortality rates continue to be unacceptably high [2, 24].

Bone marrow-derived cells penetrate the tumor and differentiate into macrophages termed tumor-associated macrophages (TAMs), which are the main component of tumor-infiltrating leukocytes [49]. Most TAMs not only lose the ability to combat tumor progression but also support tumor cell growth and metastasis [3, 40]. TAMs help to build an immune dysfunctional microenvironment in tumors by secreting many immunosuppressive cytokines [5, 26]. Furthermore, as a major source of PD-L1, TAMs inhibit cytotoxic T cell infiltration and function, which drives undesirable resistance to neoadjuvant immunotherapy [33]. In tumors, TAMs predominantly polarize into the pro-tumoral M2 phenotype [32, 48] and a high M2/M1 ratio is an independent prognostic factor in many cancers, especially melanoma [12, 34, 48]. Therefore, it is necessary to describe molecular characteristics combining patients’ M2-like TAMs infiltration and to determine the key regulatory factors of M2-like TAM polarization.

To provide new insights into the molecular features of M2-like TAM infiltration in patients with melanoma, we identified two distinct clusters (Cluster 1 and Cluster 2) based on the gene module most positively correlated with M2-like TAM infiltration in The Cancer Genome Atlas skin cutaneous melanoma (TCGA-SKCM) dataset. Then, we investigated the differences in prognosis, multi-omics, and functional enrichment between the two clusters. Next, we constructed a prognostic model according to the differentially expressed genes (DEGs) of the two clusters and compared the prognosis, immune cell infiltration, immune-related gene profile, and immunotherapy response in the high- and low-risk groups.

Subsequently, *VARS1* was characterized as the hub gene of the module most associated with M2-like TAM infiltration, which suggested that *VARS1* is linked to TAM polarization and could be defined as a new potential target in melanoma progression. VARS1 is a member of the aminoacyl-tRNA synthetases (ARSs) and its primary function is to link valines to their corresponding tRNAs in protein synthesis [28]. VARS1 mainly plays an important role in progressive brain disease [14]. Walbrecq et al. proved that hypoxia induced VARS1-bearing extracellular vesicle secretion by melanoma, which correlated with worse melanoma outcomes [60]. Nevertheless, the role of VARS1 in melanoma remains unclear.

Our study demonstrates that VARS1 expression was negatively correlated with the immune-related signaling pathways and the infiltration of antitumor cells such as CD8 T cells but was positively correlated with the accumulation of M2-like TAMs. VARS1 overexpression promoted M2-like macrophage polarization and melanoma cell migration and invasion in vitro, while knockdown of VARS1 decreased melanoma cell migration and invasion. VARS1 was inversely correlated with several immune checkpoint genes and could be a predictive biomarker of anti-PD-1 immunotherapy response. Furthermore, pan-cancer analysis revealed that VARS1 correlated negatively with CD8 T cell infiltration in most cancers and demonstrated unfavorable prognostic value in several cancers.

## Materials and methods

### Dataset source and preprocessing

The analyses involved patients from four SKCM cohorts (GSE65904, GSE98394, GSE78220, GSE91061) and TCGA-SKCM. Patients without survival information and RNA sequencing (RNA-seq) data were excluded from the analysis. For the Gene Expression Omnibus (GEO) dataset, related clinical data and transcriptome expression data were downloaded using the R GEOquery package [8] and the related GEO datasets were merged using the ComBat algorithm [31]. Transcriptome FPKM (fragments per kilobase transcript per million fragments) value and clinical data were downloaded from the Genomic Data Commons (GDC, https://portal.gdc.cancer.gov/) using the R TCGAbiolinks package [7]. The FPKM values were transformed to TPM (transcripts per million) values for subsequent analyses.

### Weighted gene co-expression network analysis (WGCNA)

We constructed mRNA co-expression networks in TCGA-SKCM dataset using the R WGCNA package [29]. First, the Pearson correlation coefficient between each pair of genes was calculated to obtain a similarity matrix. WGCNA converted the similarity matrix to an adjacency matrix using a power function. Among all soft thresholds (β) with R2 > 0.9, we chose the automatic value β (β = 5) returned by the WGCNA pickSoftThreshold function. As recommended by the WGCNA guidelines, 0.25 was chosen as the network merge height. We used default settings for other WGCNA parameters.

### M2-like TAM infiltration-related cluster acquisition

We selected the module associated with the infiltration of M2-like TAMs and CD8 T cells and the genes in this module underwent univariate Cox regression analysis. Then, the 125 genes associated with survival in univariate analysis (p < 0.05) were entered into the R ConsensusClusterPlus package [62] to perform consensus clustering for TCGA-SKCM patients. The optimal K value was identified as 2 based on the result of the cluster consensus value and cumulative distribution function.

### Development of the M2-like TAM-related prognostic model

The DEGs of two clusters with a false discovery rate (FDR) < 0.05 were identified by the R DESeq2 package [36]. Then, the 10,269 DEGs underwent univariate Cox analysis in TCGA dataset and yielded 3390 progression-associated genes (p < 0.05). Further reduction of candidate genes using lasso (least absolute shrinkage and selection operator) logistic regression with 10-fold cross-validation was performed via the R glmnet package [13]. Then, the genes were filtered further using a multivariate proportional hazard regression model (using both stepwise regression). The risk score was calculated as follows: 0.323×ATP13A5 + 0.465×C1orf105 + 0.195×TM6SF2 + 0.151×HEYL + 0.146×PTK6 + 0.065×KIT + 0.049×ENTHD1–0.209×SLC18A1–0.201×ZMAT1–0.158×CD14. The TCGA and validation cohort risk scores used the same model score threshold. Patients were stratified into low- and high-risk groups based on the median risk score cut-off and the differences in overall survival (OS) were compared using the R survival package [56]. The area under the curve (AUC) was calculated with the R timeROC package [35] to evaluate the accuracy of the prognostic model.

### Functional enrichment analysis and estimation of immune cell infiltration

Gene set variation analysis (GSVA) and gene set enrichment analysis (GSEA) were performed with the gsva [20] and clusterProfiler [65] packages in R, respectively. The gene sets for GSVA and GSEA were downloaded from the Molecular Signatures Database (MSigDB) v7.4 database. Immune cell infiltration was quantified using the CIBERSORT algorithm [47] based on the TPM value of TCGA-SKCM patients.

### Analysis of genomic alterations

Somatic mutations and somatic copy number alterations (CNAs) were downloaded from GDC using the R TCGAbiolinks package. The somatic mutations and CNAs (GISTIC output) data were visualized using the R maftools package [41]. The significant CNA amplifications and deletions were identified by GISTIC 2.0 [42]. The methylation data of TCGA patients were downloaded from the GDC portal. Differentially methylated CpGs between Cluster 1 and Cluster 2 were examined with the t-test. CpGs in chromosomes X and Y were excluded from the analysis. CpGs with FDR < 0.05 were characterized as differentially methylated CpGs.

### Protein–protein interaction (PPI) network construction and hub gene identification

The STRING database (v.11.5) was used to establish PPIs between genes in the WGCNA module with a confidence level of 0.4, and the interaction network was visualized using Cytoscape. The hub genes of the WGCNA module were screened with the Closeness, Stress, and Radiality algorithms of the cytoHubba plugin [6] in Cytoscape.

### Cell culture and transfection

We used SK-MEL-28 (ATCC, Cellcook Biotechnology, Guangzhou, China), A375 (ATCC, Cellcook Biotechnology, Guangzhou, China), and THP1 cells (ATCC, Cellcook Biotechnology, Guangzhou, China) for in vitro experiments. A375 and SK-MEL-28 cells were maintained in Dulbecco’s modified Eagle’s medium (DMEM) supplemented with 10% fetal bovine serum (FBS) and 1% penicillin-streptomycin (all from Gibco, Carlsbad, CA, USA). The THP1 cells were cultured in RPMI 1640 medium containing 10% FBS, 1% penicillin-streptomycin, 2 mM glutamine, 10 mM HEPES, and 1× non-essential amino acids (all from Gibco).

The VARS1 overexpression (pCR4-TOPO-VARS1) and control vector plasmids were purchased from Miaoling Company (Miaoling, Wuhan, China) and the small interfering RNAs (siRNAs) targeting VARS1 and the siRNA control were purchased from RiboBio (Guangzhou, China). The sequences of the *VARS1*-targeting siRNAs were as follows: GGAAACGCTCCCTGTCACAAA (VARS1 siRNA1) and GCCGGATCTGGAATAATGTGA (VARS1 siRNA2). For transient transfection, A375 and SK-MEL-28 cells were transfected with overexpression plasmid or siRNAs, respectively, using transfection reagents (Lipofectamine 3000, Invitrogen, CA, USA) for 48 h, followed by further functional assays.

### Quantitative real-time PCR (qRT-PCR) and western blotting

Total RNA extraction and qRT-PCR were conducted as previously described [64]. The qRT-PCR forward and reverse primer sequences were as follows: (1) β-actin, CTCGCCTTTGCCGATCC and TTCTCCATGTCGTCCCAGTT; and (2) VARS1, CCGTGCTAGGAGAAGTGGTT and TCTCTGGTTTTGGTTTCTTCTCCC, respectively. The western blotting was performed as previously described (36) with primary antibodies against VARS1 (WH0007407M1, Sigma, Germany) and α-tubulin (A11126, Invitrogen, CA, USA).

### Transwell migration and invasion assays

The migration and invasion assays were performed as previously described [64]. After cleaning the cells on the top of the insert, cells growing through the porous membrane were photographed with an inverted light microscope (×100). The relative numbers of migrating and invasive cells were calculated using ImageJ (ImageJ National Institutes of Health, USA).

### Flow cytometry

THP1 cells were treated with 320 nM phorbol-12-myristate-13-acetate (PMA) for 6 h and differentiated into macrophages, then maintained in the medium with PMA for 16 h to generate M0 cells as described before [17, 37, 52, 63]. To analyze the influence of VARS1 on macrophage polarization, we collected the culture supernatants of VARS1-overexpressing A375 cells at 24 h. For the CM collection method, we first seeded equal numbers (1 million cells) of VARS1-overexpressed and control cells separately in 100 mm tissue culture dishes with complete medium. When cells have grown to 70–80% confluency, replace the medium with fresh serum-free medium. After 24 h of cell culture, CM was collected and passed through a 0.22 μm filter (Millipore). Then we added the supernatant to THP-1 cell culture medium and continue to culture M0 THP1 cells. After 4 days, the THP1 cells were harvested and stained with CD86 (#374,202, BioLegend) and CD206 (#321,102, BioLegend). After 45-min incubation on ice, the cells were washed three times with phosphate-buffered saline (PBS) buffer and resuspended in fluorescence-activated cell sorting (FACS) buffer (2% FBS in PBS buffer) for flow cytometric analysis.

### Analysis of the immunotherapy response

We integrated two datasets of patients with melanoma treated with anti-PD-1 (GSE78220 and GSE91061). Further analyses were performed only on treatment-naïve patients. Then, the immunotherapy response was predicted using the SubMap online tool [30].

### Statistical analysis

Survival differences between groups were assessed using Kaplan-Meier curves and log-rank tests. Prognostic factors were determined with univariate and multivariate Cox regression analyses. Correlation coefficients were calculated by Pearson and Spearman correlation analyses. Normal and non-normal variables were compared using the unpaired Student t-test and the Mann-Whitney U test, respectively. One-way analysis of variance and the Kruskal-Wallis test were used as parametric and nonparametric methods, respectively, for comparing > 2 groups. Genes with differential mutations and differential copy number losses and gains were examined with chi-square and Fisher’s exact texts. The statistical analysis was performed using R software and values represent the mean ± standard deviation. P < 0.05 was considered statistically significant.

## Results

### Identification of M2-like TAM-related cluster

**Fig. 1:**
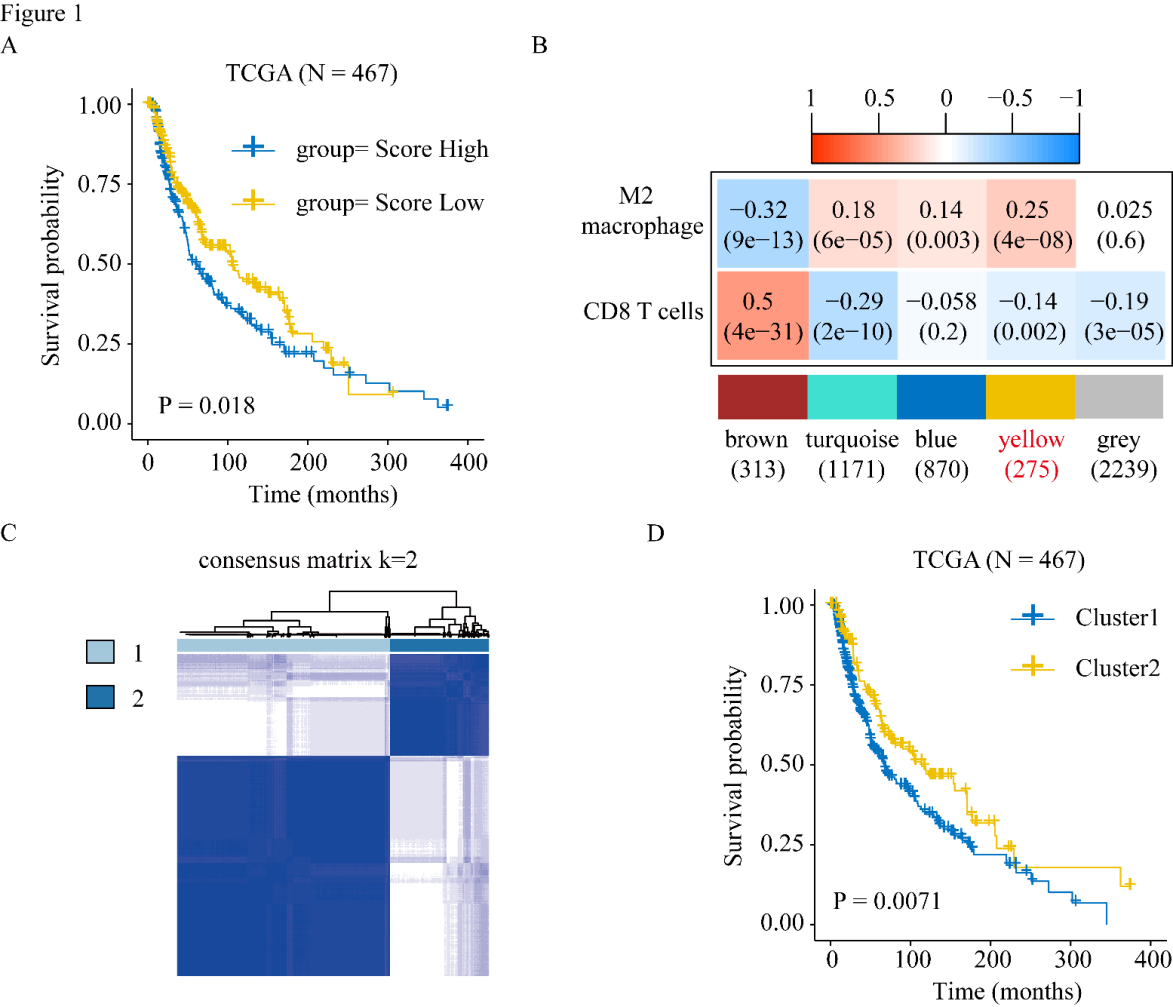
Identification of M2-like TAMs related cluster. (A) Kaplan–Meier analysis showing the correlations between M2-like TAMs infiltration and overall survival (OS) in TCGA SKCM cohorts. Patients were grouped into “high” or “low” groups based on the median CIBERSORT-based M2 macrophages score. (B) Weighted correlation network analysis (WGCNA) identifies M2-like TAMs and CD8 T cells infiltration correlated modules. (C) Consensus clustering showed that 2 clusters were most stable. (D) Kaplan-Meier survival analysis was performed to analyze the difference in overall survival (OS) of the two clusters

First, we used the CIBERSORT algorithm to assess the fraction of immune cell infiltration in patients. In TCGA and GSE98394 datasets, patients with a higher proportion of M2 macrophage infiltration had worse prognosis (Fig. 1 A and Figure S1A). Considering that more M2 macrophages appeared to be associated with poorer prognosis and CD8 T cell infiltration, we performed WGCNA to detect the module related to CD8 T cell and M2 macrophage infiltration (Figure S1D). We select the soft threshold power β = 5 (scale-free R2 = 0.90) to construct a scale-free network (Figures S1B, S1C).

The heatmap demonstrates that the yellow module was negatively and positively correlated with the infiltration of CD8 + T cells and M2 macrophages, respectively, in TCGA-SKCM (Fig. 1B). We used the genes in the yellow module and survival data in TCGA-SKCM dataset to perform univariate Cox regression analysis, and 125 genes were associated with OS in TCGA-SKCM. We used the R ConsensusClusterPlus package for consistent clustering in TCGA-SKCM dataset based on the 125 prognostic genes and identified two clusters: Cluster 1 (319 cases) and Cluster 2 (148 cases) (Fig. 1 C and Figure S1E, S1F). Principal component analysis also suggested that these two populations were distinct groups (Figure S1G). Cluster 1 had worse OS outcomes than Cluster 2 (log-rank p = 0.0071, Fig. 1D).

### Functional and multi-omics analyses

**Fig. 2:**
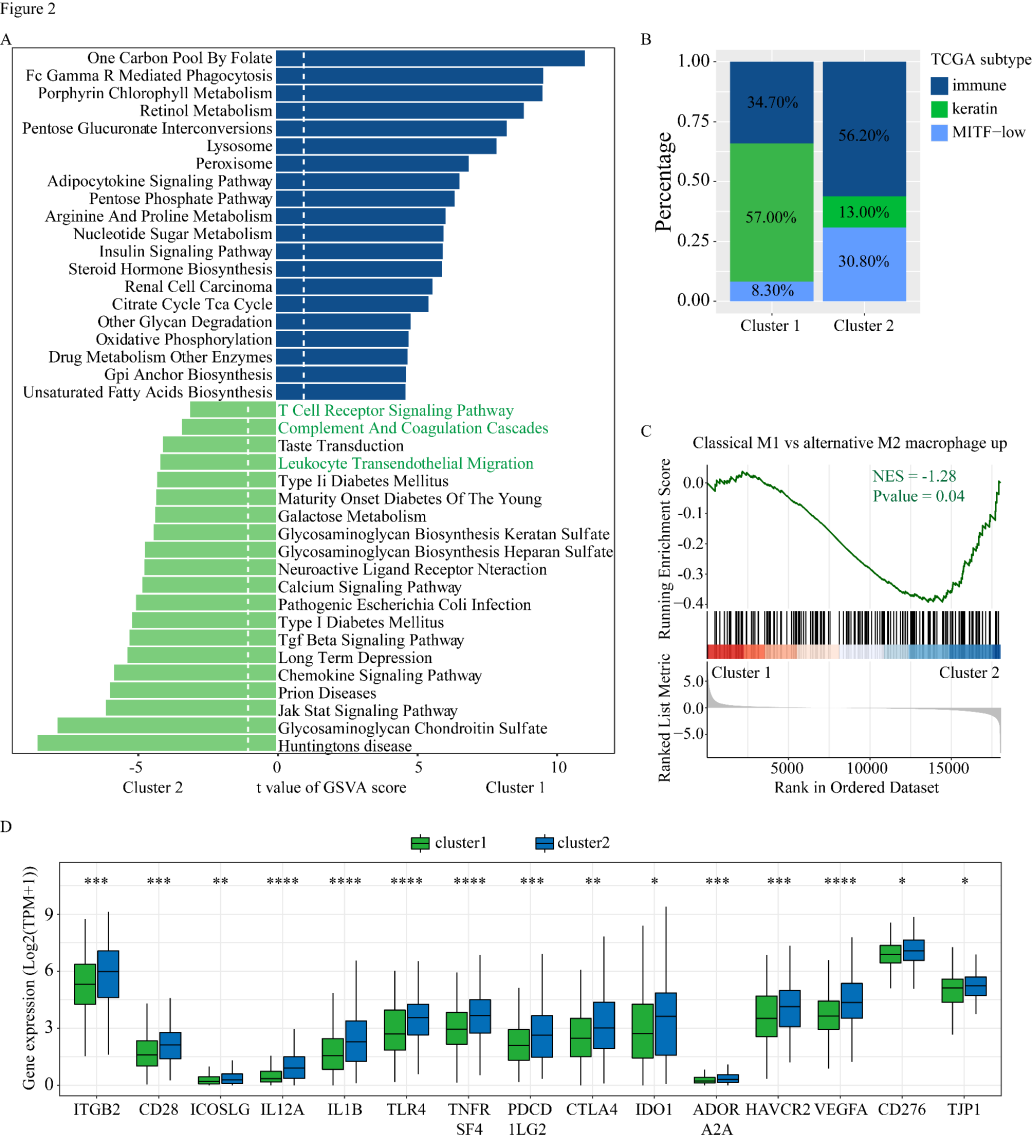
Functional analysis and differential expression analysis of two clusters. (A) The top 20 enriched KEGG pathways for each cluster were explored by GSVA analysis. (B) Percentage of patients with different TCGA melanoma subtypes in different clusters. (C) GSEA analysis showing that the correlation of clusters with M2 macrophage gene sets. (D) The differences in expression of immune checkpoint-related genes between the two clusters. ‘*’ represents p-value ≤ 0.05, ‘**’ represents p-value ≤ 0.01, ‘***’ represents p-value ≤ 0.001, N.S indicates not significant (p > 0.05)

To demonstrate signaling pathway activation in each cluster, we calculated the GSVA enrichment scores using Kyoto Encyclopedia of Genes and Genomes (KEGG) signaling pathway gene sets in MSigDB v7.4. Figure 2 A depicts the top 20 enriched pathways in each cluster. In comparison with Cluster 2, Cluster 1 was characterized by the lack of immune-related pathways, such as T cell receptor signaling pathways. A previous study divided TCGA-SKCM tumors into three subtypes [1]: (1) immune, (2) keratin, and (3) MITF-low. We found that Cluster 1 contained a higher proportion of the keratin subtype (57% vs. 13%) and a lower proportion of the immune subtype (34.7% vs. 56.2%) than Cluster 2 (Fig. 2B).

**Fig. 3:**
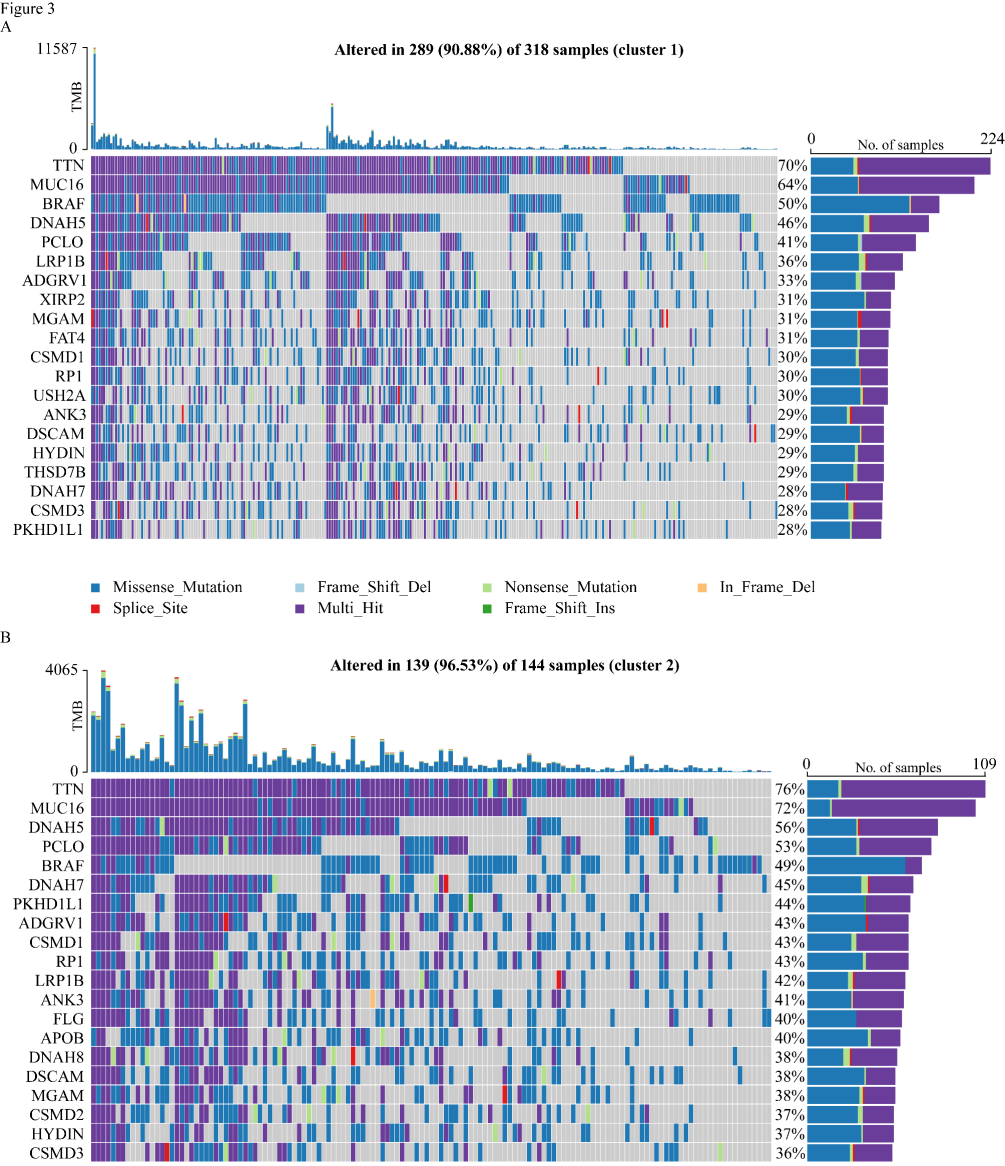
The mutation analysis of two clusters. The waterfall plot showing the top 20 genes with mutation frequency of Cluster 1 (A) and Cluster 2 (B). Each column represents an individual patient. The upper histogram is the total tumor mutation burden (TMB), and the numbers on the right are the mutation frequencies of each gene. The bar graph on the right is the proportion of each mutation type

GSEA indicated that the M2 macrophage pathway was enriched in Cluster 1 (Fig. 2 C). Examination of the differential expression of immune checkpoint genes revealed that Cluster 2 demonstrated higher immune checkpoint-related gene expression compared with Cluster 1 (Fig. 2D). To investigate mutations in each cluster, we highlighted the top 20 significantly mutated genes (SMGs) in the two clusters with a waterfall plot (Fig. 3 A, 3B). The two clusters shared most of the SMGs. However, Cluster 1 contained unique SMGs, including *XIRP2* (31%), *FAT4* (31%), *USH2A* (30%), and *ANK3* (29%) while Cluster 2 contained unique SMGs that included *FLG* (40%), *APOB* (40%), and *CSMD2* (37%).

**Fig. 4:**
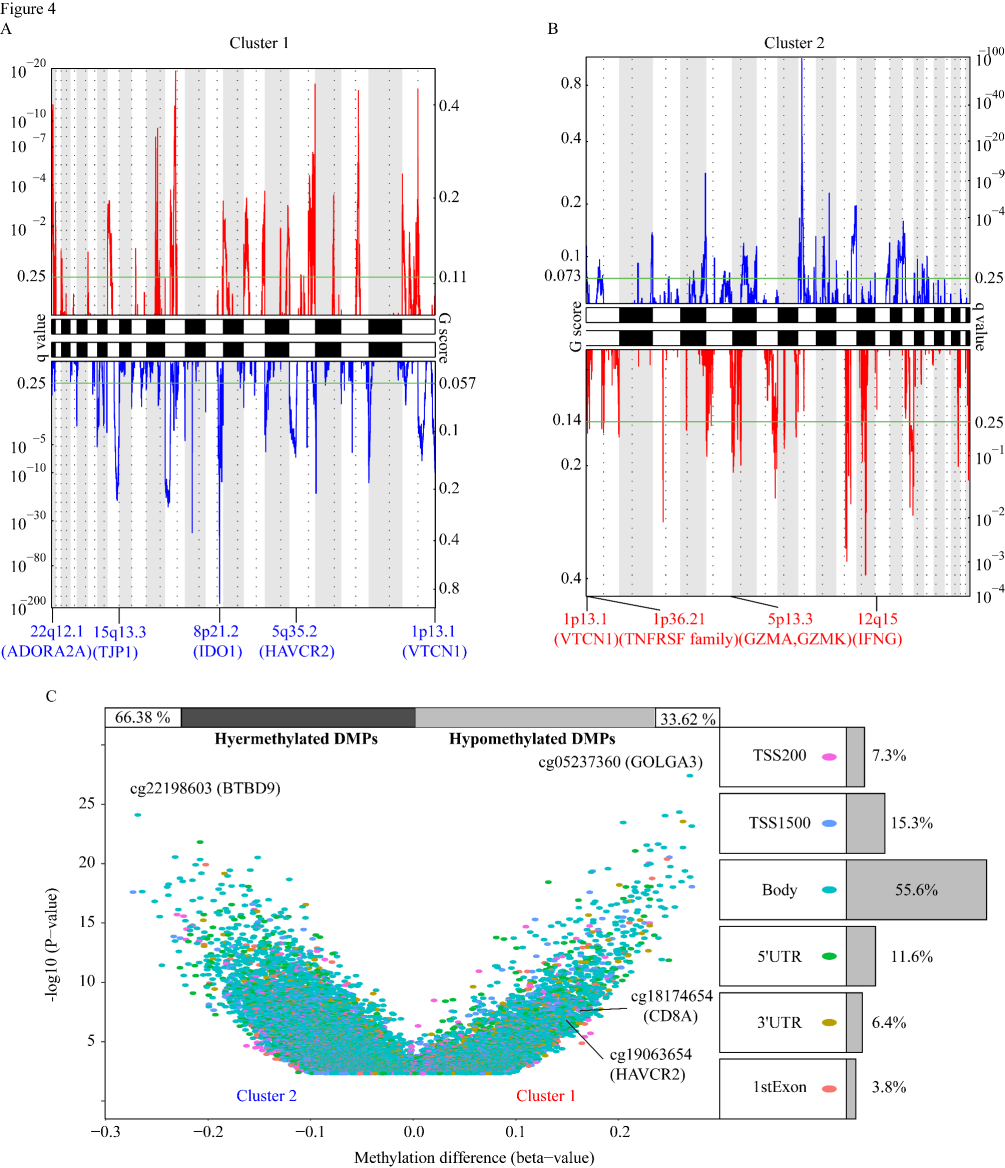
Multi-omics analysis of two clusters. GISTIC 2.0 analysis determining the statistically significant amplifications and deletions in Cluster1 (A) and Cluster2 (B). Statistically significant gains (red) and losses (blue) of chromosomal locations are shown. The q-value, which characterize statistical significance, are shown below the graph. Areas with q-values < 0.25 (green lines) are considered significantly changed. These peak regions were annotated with known immune checkpoint related genes. (C) Volcano plots show alterations in DNA methylation that are statistically significant between the two clusters. The right side shows different proportions of genomic features

A recent prospective study found that higher tumor mutation burden (TMB) is associated with better immunotherapy response [4]. Cluster 2 samples demonstrated higher TMB severity than Cluster 1 samples (Figure S2A). We used GISTIC 2.0 to analyze the somatic copy number variation (SCNV) and summarized the amplified and deleted areas of Cluster 1 and Cluster 2. Cluster 1 contained a total of 56 focal deletion peaks and 69 focal amplification peaks, while Cluster 2 contained 37 focal deletion peaks and 28 focal amplification peaks (Fig. 4 A, 4B). Examination of the frequency of immune checkpoint gene amplification or deletion in each subtype revealed that Cluster 2 contained more amplification of immune checkpoint (*VTCN1*, *TNFRSF* family) and effector T cell function genes (*GZMK*, *GZMA*, *IFNG*) while Cluster 1 had more deletions (*VTCN1*, *ADORA2A*, *TJP1*, *IDO1*, *HAVCR2*) (Fig. 4 A, 4B). We used the R ChAMP package [57] with FDR < 0.05 to analyze the methylation differences in the two clusters and obtained 28,870 differentially methylated probes (DMPs) between Cluster 1 and Cluster 2. Interestingly, *CD8A* and *HAVCR2* of Cluster 1 had increased methylation levels than that in Cluster 2 (Fig. 4 C).

### Construction of the M2 macrophage cluster-related prognostic model

**Fig. 5:**
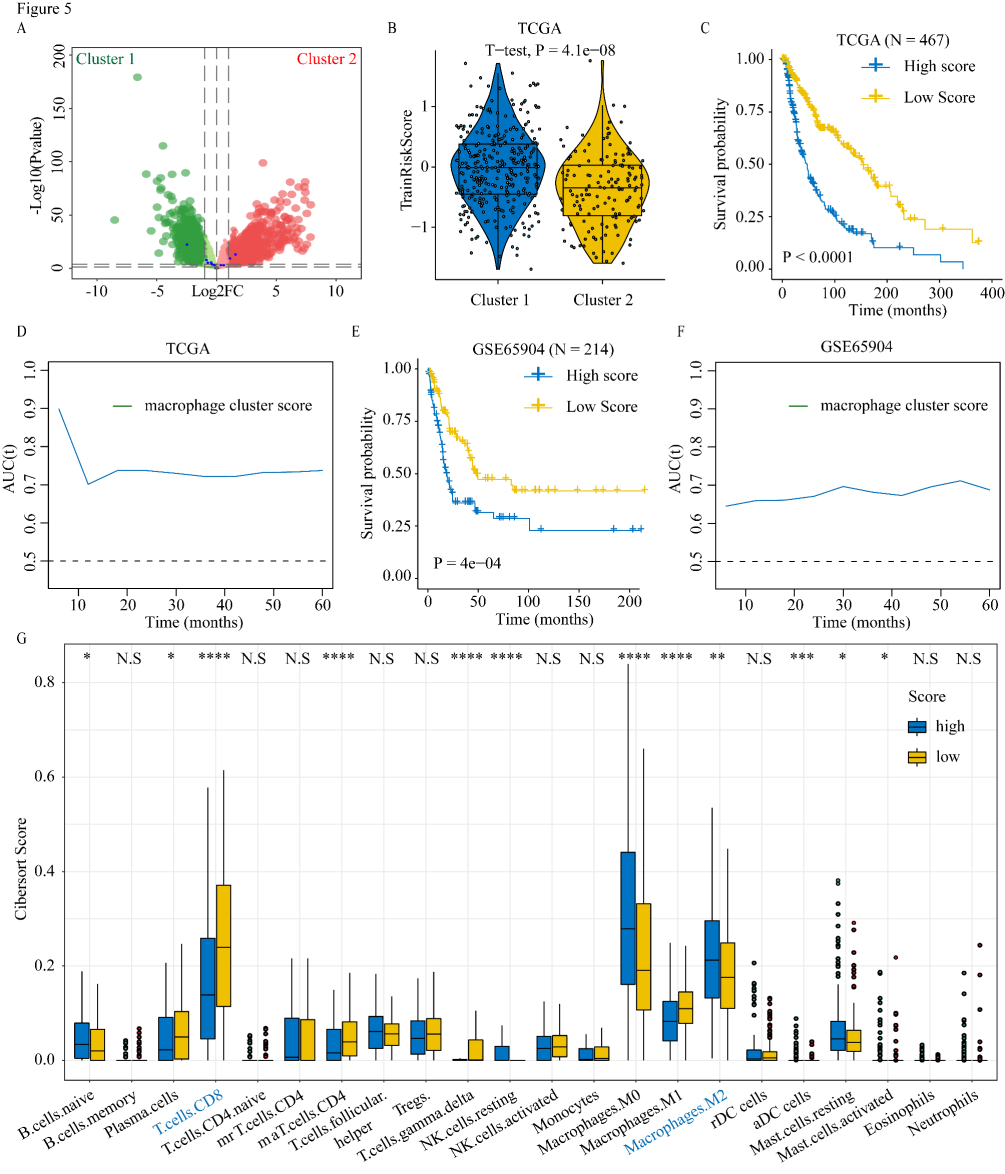
Construction of the M2 macrophage cluster Related Prognostic Model. (A) Volcano plot showing differential expressed genes in two clusters. (B) The differences in risk scores of prognostic models between two clusters. The difference in overall survival between low-risk score and high-risk score groups in TCGA melanoma cohort (C) and GSE65904 melanoma cohort (E). Patients were grouped into “high” or “low” groups based on the median risk score. Time-dependent areas under the curve (AUC) values in TCGA (D) and GSE65904 (F). (G) The comparison of the immune cells infiltration between high-risk and low-risk groups

We explored the DEGs between the two clusters to construct a prognostic model (Fig. 5 A). First, we performed univariate Cox analysis on the DEGs and obtained 3390 genes with prognostic significance. Then, we performed lasso regression and multivariate Cox analysis based on the 3390 genes to construct a prognostic model in TCGA-SKCM dataset (Figure S2B, S2C). The risk score was calculated as follows: 0.323×ATP13A5 + 0.465×C1orf105 + 0.195×TM6SF2 + 0.151×HEYL + 0.146×PTK6 + 0.065×KIT + 0.049×ENTHD1–0.209×SLC18A1–0.201×ZMAT1–0.158×CD14. Then, TCGA-SKCM patients were divided into high- and low-risk groups based on their risk scores. Patients with higher risk scores had worse OS prognosis, and Cluster 1 patients had higher risk scores (Fig. 5B C).

Time-dependent AUC and the AUCs at 1 (0.70), 2 (0.74), 3 (0.72), and 5 (0.74) years suggested that the M2 macrophage cluster-related risk score had potential value for predicting the OS of patients with melanoma in TCGA datasets (Fig. 5D and Figure S2D). To verify the prognostic significance of the model, we used the same model score threshold to calculate the risk score in a validation cohort (GSE65904), which yielded a similar result, where patients with higher risk scores had worse OS, and the risk score had prognostic value (Fig. 5E F and Figure S2E). The risk score was identified as an independent prognostic factor in both TCGA and GSE65904 datasets (Table S1).

### Differences in immune cell infiltration and immune gene expression between high- and low-risk groups

**Fig. 6:**
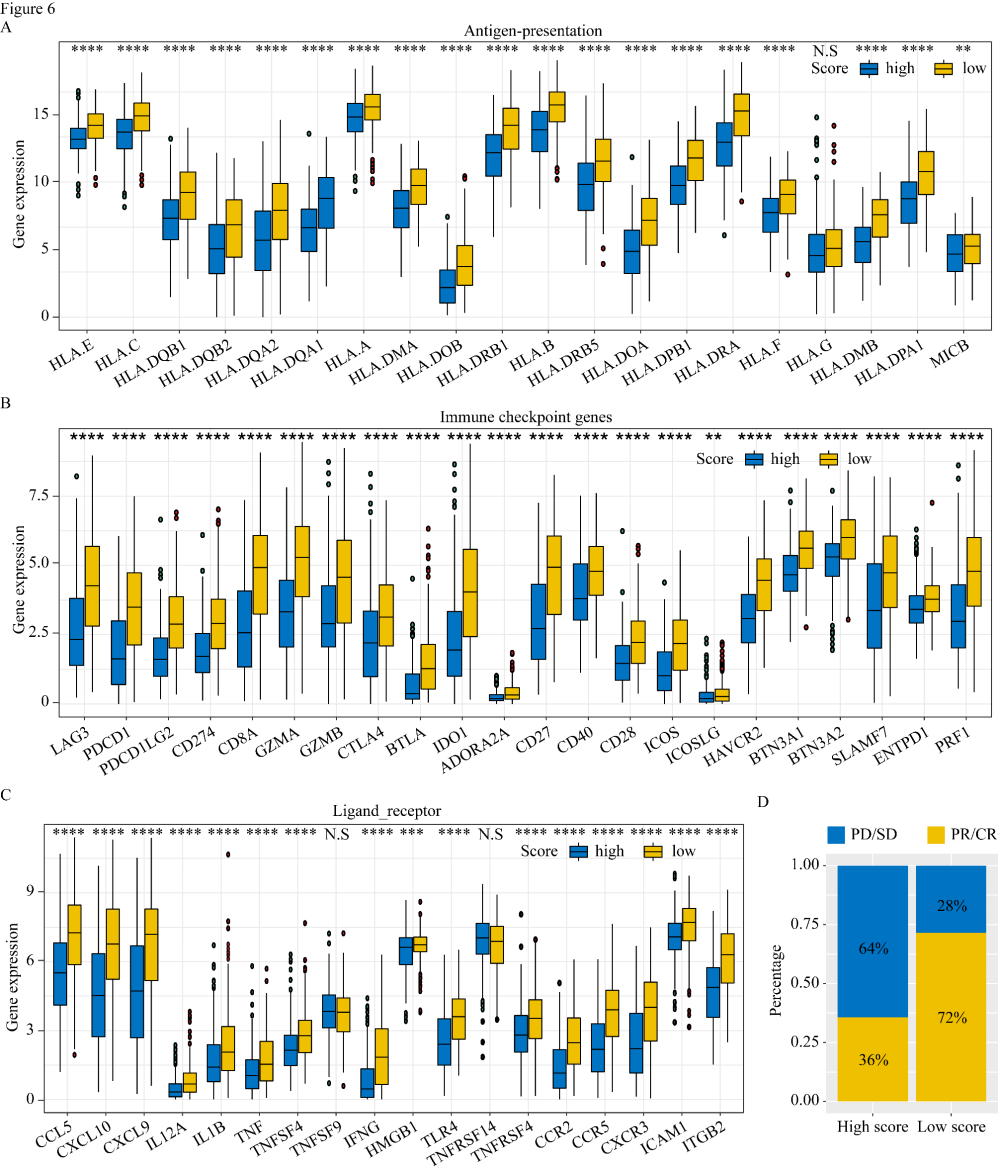
Differences in immune check point related gene and response to anti-PD-1 immunotherapy between high and low risk groups. Boxplots displayed the differences in the expression of antigen presentation (A), immune check point genes (B) and several ligand-receptor (C) in TCGA melanoma cohort. ‘*’ represents p-value ≤ 0.05, ‘**’ represents p-value ≤ 0.01, ‘***’ represents p-value ≤ 0.001, N.S indicates not significant (p > 0.05). (D) The proportion of patients with response to anti-PD-1 immunotherapy in different risk group. SD: stable disease; PD: progressive disease; CR: complete response; PR: partial response

The risk score played an important role in melanoma progression. To assess the influence of the M2 macrophage cluster-related risk score on the tumor microenvironment (TME), we compared the immune cell infiltration between the high and low score groups. Patients with high risk scores had increased M2 macrophage infiltration and decreased CD8 T cell infiltration compared to patients with low risk scores (Fig. 5G). We also explored differences in the expression of *HLA* family genes and immune checkpoint markers in the high and low risk score groups in TCGA and GEO datasets. The high risk score group had significantly increased expression of the antigen-presentation and immune checkpoint-related genes in comparison to the low risk score group of TCGA datasets (Fig. 6 A–C). Consistent with these results, analysis of GSE65904 sample data yielded similar results (Figure S3A–C). Furthermore, we applied our M2 macrophage cluster-related model to the merged datasets (GSE78220 and GSE91061) with available immunotherapy outcomes and examined the risk score of melanoma patients. To further observe the different response to immunotherapy in high risk score and low risk score groups, we found that patients with high risk score had higher proportion of non-responders to immunotherapy compared to patients with low risk score (64% vs. 28%). (Fig. 6D)

### VARS1 as a hub gene of the yellow module and its role in melanoma progression and macrophage polarization

**Fig. 7:**
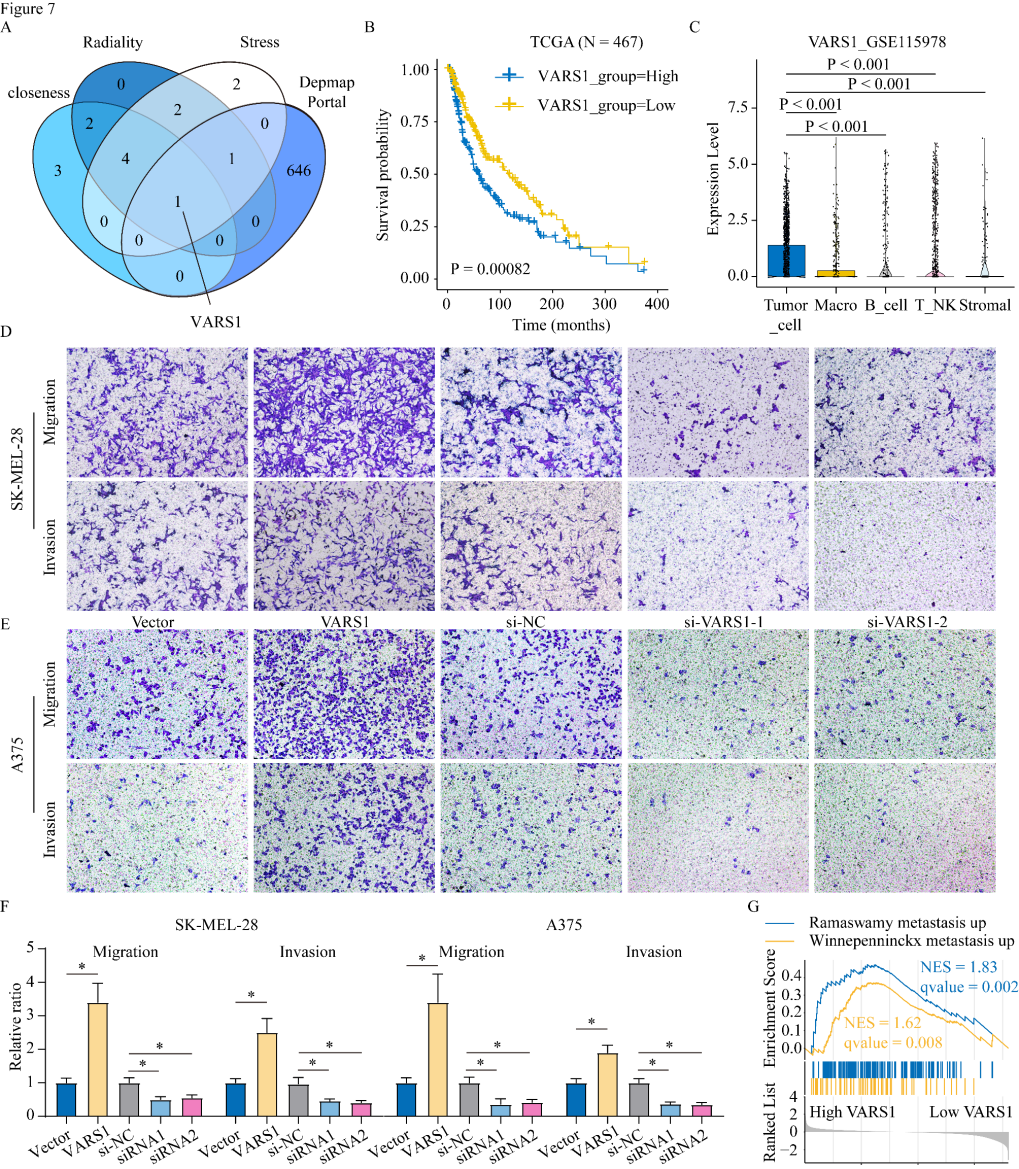
VARS1 as a Hub Gene and its Role in Melanoma progression. (A) Venn diagram showing the intersection of hub genes of the M2 infiltration-related module and genes critical for the growth of melanoma human cell lines in the DepMap database. (B) Overall survival of TCGA melanoma patients with high and low VARS1 expression measured by Kaplan–Meier analysis. Patients were grouped into “high” or “low” groups based on the median expression of VARS1. (C) Analysis of VARS1 expression in various cell types in single-cell sequencing datasets. (D-F) Overexpressing VARS1 promoted migration and invasion abilities in SK-MEL-28 cells and A375 cells, while silencing VARS1 suppressed the abilities. ‘*’ represents p-value ≤ 0.05. (G) GSEA analysis showing that the correlation of VARS1 expression with metastasis-related gene sets

We explored the hub genes in the yellow module. We used the 275 genes in the yellow module to construct a PPI network based on the STRING database results. Then, the top hub genes were determined via the Closeness, Stress, and Radiality algorithms in the Cytoscape cytoHubba plugin (Figure S4). The hub gene essential for melanoma cell growth was determined with DepMap (https://depmap.org/portal/download/), a CRISPR-based database for genome-wide loss-of-function screening. Only *VARS1* was identified by intersecting the gene sets obtained from these four methods (Fig. 7 A). In TCGA dataset, high *VARS1* expression correlated with shorter OS (Fig. 7B). Furthermore, we explored which cell type mainly expressed VARS1 in melanoma. The result of single-cell RNA-seq of the GSE115978 dataset demonstrated that VARS1 was expressed predominantly in tumor cells but not in stromal and immune cells (Fig. 7 C). Additionally, high risk score patients had higher *VARS1* expression levels than low risk score patients (Figure S5A).

We also examined whether *VARS1* played an important role in melanoma progression and constructed *VARS1*-overexpressing and *VARS1* knockdown A375 and SK-MEL-28 cell lines (Figure S5B). *VARS1* overexpression promoted the migration and invasive ability of the cells while *VARS1* suppression significantly decreased it (Fig. 7D–F). GSEA indicated that high *VARS1* levels positively correlated with the metastasis-related pathway in TCGA-SKCM dataset (Fig. 7G). Furthermore, a search of the Human Protein Atlas (HPA) database [58, 59] showed that VARS1 expression was increased in primary melanoma compared to normal skin tissue, and further increased in metastatic melanoma (Figure S5D).

### VARS1 negatively correlated with immune infiltration and induced M2 macrophage polarization

**Fig. 8:**
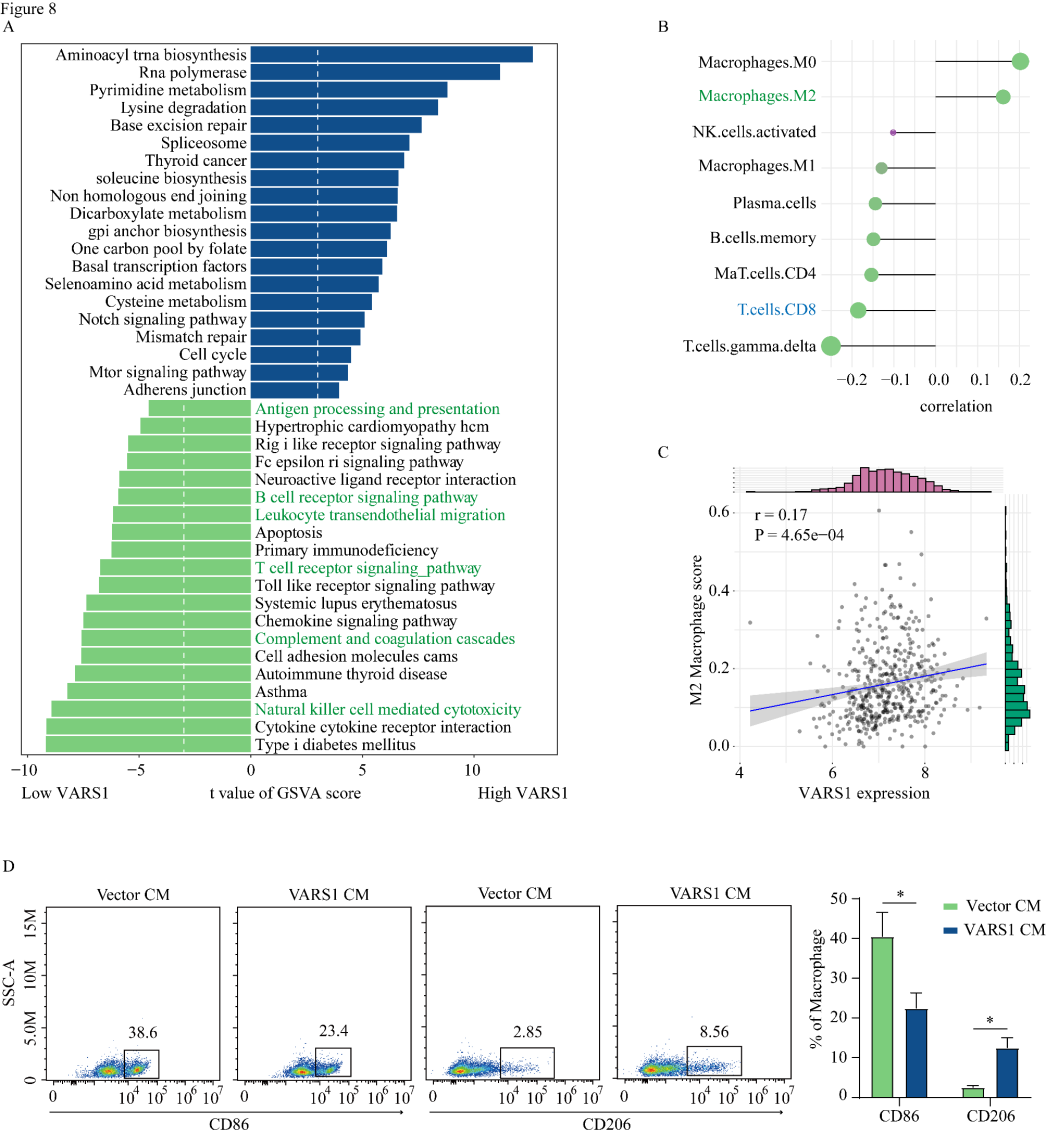
The role of VARS1 in immune cell infiltration and macrophage polarization. (A) KEGG pathway enrichment scores between high and low VARS1 expression groups analyzed using GSVA and showing the top 20 differential pathways. Patients were grouped into “high” or “low” groups based on the median expression of VARS1. (B) The graph shows the correlation between VARS1 expression and immune cell infiltration based on the output of Cibersort analysis. The correlation coefficients were calculated by the Spearman rank correlation test. (C) The correlation of VARS1 expression and M2-like TAMs infiltration. (D) THP-1 cells were treated with supernatant of VARS1-overexpressing A375 cells and then the polarization of THP-1 was analyzed by flow cytometry. ‘*’ represents p-value ≤ 0.05

To investigate the VARS1-related pathways, we divided TCGA-SKCM dataset patients into two groups based on the median *VARS1* gene expression. GSVA of the KEGG pathways revealed that the immune-related pathways, such as the T cell receptor pathway, were enriched in patients with low *VARS1* expression, while tumor growth pathways such as the cell cycle pathway and the mTOR pathway were enriched in patients with high *VARS1* expression (Fig. 8 A).

We examined the correlation between *VARS1* expression and the CIBERSORT immune cell infiltration score. *VARS1* expression positively correlated with intratumoral M2 macrophage infiltration and negatively correlated with M1 macrophage and CD8 T cell infiltration (Fig. 8B C). To elucidate the role of VARS1 in M2 macrophage polarization, THP1 cells were treated with the supernatant of A375 cells line overexpressing *VARS1* (VARS1-A375) and A375 vector (vector-A375) cell lines and detected the M1 and M2 macrophage markers. Flow cytometry revealed a 3-fold increase in the expression of the M2 macrophage marker CD206 in THP1 cells treated with VARS1-A375 supernatants compared with those treated with vector-A375-supernatants, while the expression of CD86, an M1 macrophage marker, decreased by 15.2% (Fig. 8D). Taken together, these results indicate that VARS1 may play important roles in M2 macrophage infiltration and polarization.

### High VARS1 expression correlated with low CD8 T cell infiltration and predicted the poor clinical benefit of immune checkpoint blockade

**Fig. 9:**
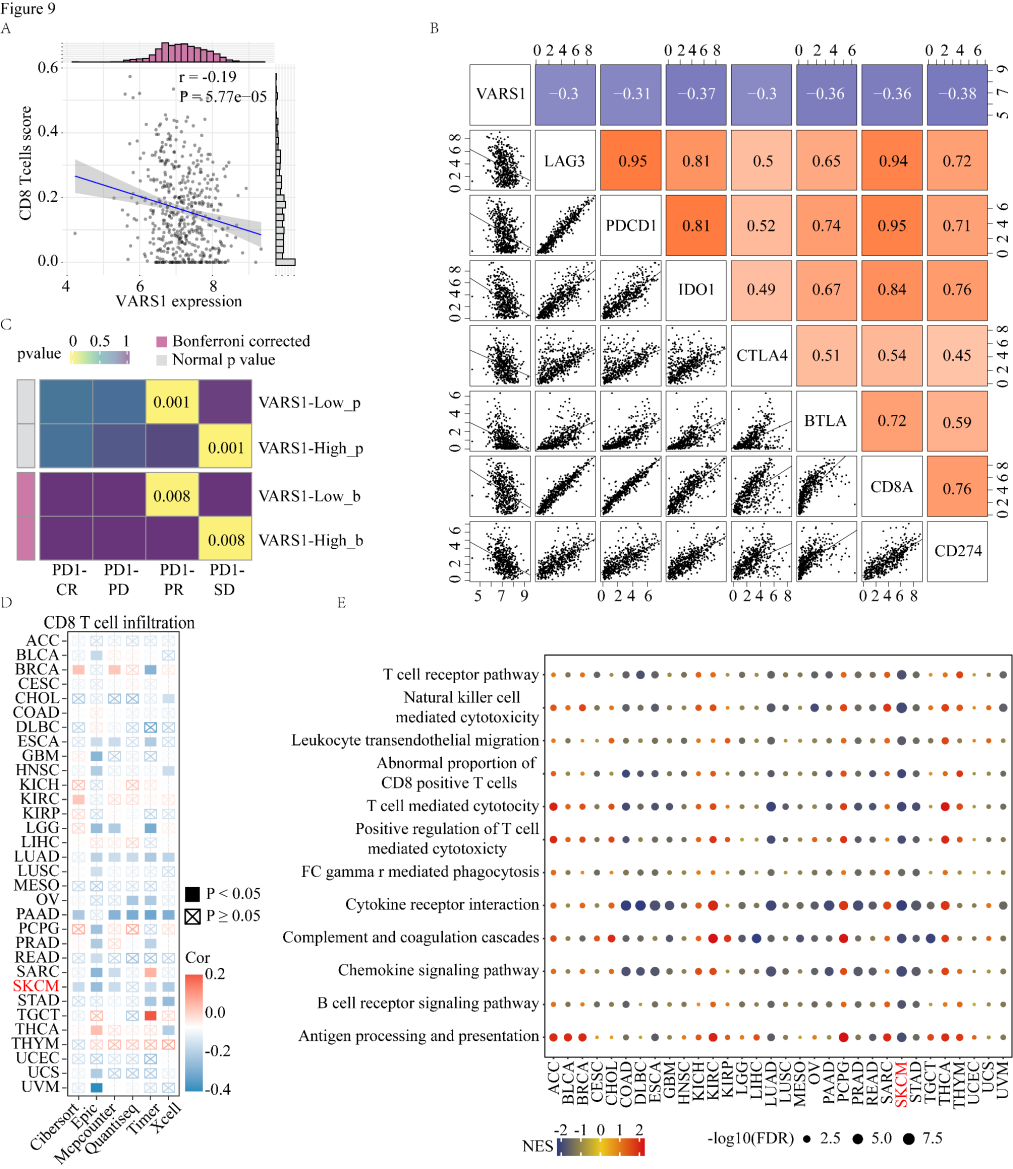
High expression of VARS1 correlates with low CD8 T cell infiltration and predict the poor clinical benefit of ICB. (A) The correlation of VARS1 expression and M2-like TAMs infiltration. (B) Correlation between the expression of VARS1 and several known immune checkpoint genes in the TCGA dataset. The correlation coefficients were calculated by the Pearson correlation test. (C) The submap tool analysis showed that VARS1 expression could predict the response to anti-PD-1 treatment. The p values obtained were adjusted by the Bonferroni method. (D) Pan-cancer analysis investigating the correlations between VARS1 expression and CD8 T cell infiltration across 32 cancer types from the TCGA dataset. The correlation coefficients were calculated by the Spearman rank correlation test. (E) Pan-cancer GSEA analysis for immune response related pathway between high- and low-VARS1 tumor tissues. NES, normalized enrichment score; FDR, false discovery rate

High *VARS1* expression correlated negatively with CD8 T cell infiltration in TCGA-SKCM dataset (Fig. 9 A). The expression of many immune checkpoint genes was negatively associated with *VARS1* expression in both TCGA and GSE65904 datasets (Fig. 9B and Figure S6A). Previous studies have shown that TGF-β1 is involved in PD-1 immunotherapy resistance and M2 macrophage polarization [11, 66]. Here, the enzyme-linked immunosorbent assay demonstrated that the supernatant of VARS1-overexpressing cells had significantly increased TGF-β1 concentrations compared to that of vector cells (Figure S5C). We performed SubMap analysis to assess the anti-PD-1 immunotherapy response in high- and low-*VARS1* expression patients with melanoma. The results demonstrated that low *VARS1* expression predicted partial response (PR) to anti-PD-1 immunotherapy whereas high *VARS1* expression predicted resistance (SD) to anti-PD-1 immunotherapy (Fig. 9 C). To explore the suppressive role of VARS1 in immune regulation, we used different algorithms to investigate the correlation between *VARS1* gene expression and CD8 T cell infiltration in Pan-TCGA datasets. The heatmap showed that *VARS1* gene expression and CD8 T cell infiltration were inversely correlated in most cancers (Fig. 9D).

GSEA indicated that many immune-related pathways, such as the T cell-mediated cytotoxicity pathway, were enriched in the patients with high *VARS1* expression in 70% of cancer types (Fig. 9E). Finally, we evaluated the association between *VARS1* and OS across 33 cancer types. High *VARS1* expression was correlated with poorer survival in six cancer types (Figure S6B), including KICH (hazard ratio [HR] = 2.80), MESO (HR = 1.74), SKCM (HR = 1.32), SARC (HR = 2.25), LAML (HR = 1.69), and CESC (HR = 1.49) and with better survival in READ (HR = 0.47). These results suggest that VARS1 may have predictive value for patient prognosis and PD-1 immunotherapy efficacy.

## Discussion

Melanoma has been recognized as the most aggressive type of skin cancer and is particularly responsive to immunotherapy such as immune checkpoint blockade with CTLA4 and PD-1 antagonists [38]. Immunotherapy can improve patient outcomes obviously, especially for patients with stage IV melanoma, but the mortality rates would become quite high once patients develop immunotherapy resistance [2, 53, 54]. Nevertheless, the goal of addressing and predicting immunotherapy response in melanoma has been reached. Considering that numerous studies have demonstrated the importance of TAMs in clinical outcome and immunotherapy resistance in melanoma, we applied WGCNA to identify a M2-like TAM module in melanoma for the first time and examine the reliability of M2-like TAMs as a prognostic marker in melanoma and in predicting immunotherapy response.

Recent studies have demonstrated the prognostic importance of TAMs in various cancers. The presence of TAMs, mainly M2-like TAMs, is not only correlated with poor outcome in various tumors, but is also associated with the generation of an immunosuppressive TME [16, 22, 46]. As an important source of inflammatory cytokines and growth factors, M2-like TAMs support angiogenesis, which results in the promotion of tumor cell proliferation and survival [9, 21, 51]. A previous study reported that TAM-derived VEGFA enhanced vascular permeability, thereby facilitating cancer cell intravasation and metastasis [19]. Moreover, M2-like TAMs express PD-L1, a major negative regulatory ligand suppressing cytotoxic T lymphocyte (CTL) activation in the TME. In some cancers, M2-like TAM-derived PD-L1 is more effective than cancer cell-derived PD-L1 for suppressing CTL function [27, 50]. Recent studies have demonstrated that M2-like TAM-derived factors, such as interleukin (IL)-6, IL-10, and milk fat globule-epidermal growth factor VIII (MFG-E8), can suppress naïve T cell proliferation, promote carboplatin resistance, and enhance tumor growth [23, 39, 61]. Furthermore, depleting or downregulating M2-TAMs suppressed tumor growth by inactivating CCL2 and/or CCR2 signaling [55]. However, a M2-like TAM-related prognostic model in melanoma has not been explored.

Based on the importance of M2-like TAMs to clinical outcome and the immunosuppressive TME, we inferred that a gene module associated with M2-like TAMs in melanoma could be applied to establish a prognostic model that could provide predictive value in clinical outcome and immunotherapy response in melanoma. We first validated that the high score of M2-like macrophages is significantly associated with poorer survival in TCGA and GSE98394 datasets. To examine the reliability of M2-like TAMs as a prognostic marker in melanoma, two clusters were grouped by genes in a M2-like TAM-related module and demonstrated different OS and clinical features.

With poorer OS, Cluster 1 was characterized by enrichment of the M2 macrophage pathway and the lack of immune response pathways, such as the T cell receptor signaling pathway, complement and coagulation cascades, and leukocyte transendothelial migration. The activation of these immune response pathways is associated with good immunotherapy response and good clinical outcome [10, 15, 18, 54], indicating that the lack of immune response pathways was one of the major leading causes of the poorer outcome in Cluster 1 as compared with Cluster 2. Furthermore, the transcriptomic classification of melanoma includes the immune, keratin, and MITF-low subtypes. Compared with Cluster 2, Cluster 1 had a lower proportion of immune-subtype melanoma, which is associated with overexpression of the immune-related genes and more favorable post-accession survival. Moreover, Cluster 1 also contained a higher proportion of the keratin subtype, which exhibits worse outcome when compared with the immune and MITF-low subtypes.

As an emerging predictive biomarker of cancer immunotherapy, elevated TMB can be associated with increased clinical benefit from immune checkpoint blockade therapies [4]. Interestingly, Cluster 2 had higher TMB severity than Cluster (1) Recent studies have also shown that checkpoint blockade immunotherapy response is correlated with the immune checkpoint gene and ligand receptor expression level [45]. Cluster 2 had more amplifications of the immune checkpoint and effector T cell function genes, while Cluster 1 had more deletions of the genes. This indicated that Cluster 1 had more decreased benefit from immunotherapy compared to Cluster (2) Our results suggest that the identified M2-like TAM module is reliable for providing meaningful prognostic value in the clinical outcome and immunotherapy response in melanoma.

We further identified a M2 macrophage cluster-related prognostic model and generated a prognostic risk score based on the DEGs between the M2-like TAM-related clusters. In TCGA cohort, Cluster 1 had a significantly higher risk score than Cluster 2, and OS was significantly decreased in the high risk score group compared to the low risk score group. Moreover, a higher risk score was associated with a series of tumor immunogenic factors. In our study, the high risk score group demonstrated less CD8 + T cell infiltration and more M2 macrophage infiltration compared to the low risk score group. Previous studies have proven that inhibiting antigen presentation is associated with immune evasion. The antitumor immune response is mainly centered on antigen presentation. Our result demonstrated that the high risk score group had significantly suppressed antigen presentation compared to the low risk score group, indicating that a higher risk score was associated with lower immunotherapy response. Furthermore, our findings also demonstrate that compared with the low risk score group, the high risk score group had decreased expression of the immune checkpoint genes and the majority of ligand receptors, including CCL5, CXCL9, and IFNG. This observation prompted us to examine the prognostic value of this risk score in immunotherapy outcomes: there was a higher percentage of SD/progressive disease in high-risk patients than in low-risk patients. Hence, the risk score based on the M2-like TAM-related prognostic model represented an independent prognosticator of OS and immunotherapy response in melanoma.

With the aim of identifying a potential biomarker for predicting OS and immunotherapy response in melanoma, we identified the top hub genes in the specific M2-like TAM module via three different algorithms. Interestingly, only *VARS1* was identified after intersection between these hub genes and the melanoma cell growth-related genes in the DepMap database, indicating that *VARS1* was associated with M2-like TAM polarization and melanoma tumor cell growth. Moreover, our results showed that *VARS1* was mainly expressed by tumor cells and that high *VARS1* expression was significantly associated with poor OS and the metastasis-related pathway in TCGA-SKCM dataset. As an ARS member, VARS1 plays an important role in protein synthesis. Recent studies have shown that ARSs are involved in various physiological and pathological processes, especially tumorigenesis, and could be potential biomarkers and therapeutic targets in cancer treatment [25]. However, only one study reported that VARS1-bearing extracellular vesicles were associated with worse clinical outcome in melanoma [60]. The role of VARS1 in melanoma remains unclear, which prompted our exploration of the function of VARS1 as a potential prognostic biomarker in melanoma.

Our in vitro experiments demonstrated that A375 and SK-MEL-28 cell migration and invasive ability was significantly increased after *VARS1* was overexpressed, while *VARS1* knockdown decreased it. Moreover, high *VARS1* expression was associated with low immune-related signaling pathway enrichment, low immune checkpoint expression, and low CD8 T cell infiltration and predicted anti-PD-1 immunotherapy resistance, which indicated that the upregulation of VARS1 can be associated with low immunotherapy response and poor clinical outcome in melanoma. Previous studies have also shown that the tumor-suppressing effect of the TGF-β1 signaling pathway has an essential function in poor immunotherapy response [11]. Our in vitro experiments demonstrated that VARS1 upregulated TGF-β1 expression in tumor cells and the M2 macrophage marker CD206. In addition, our analysis of the Pan-TCGA datasets supported the idea that high *VARS1* expression was correlated with poor CD8 T cell infiltration in most cancers. Taken together, our results suggest that, as the hub gene related to the M2-like macrophage module, *VARS1* exerts an immunosuppressive effect on melanoma progression and is a potential predictive biomarker of clinical outcome and immunotherapy response in melanoma, which requires further investigation in prospective studies and larger populations.

Our study has potential weaknesses. It is a retrospective study and requires a multi-center cohort study to validate the predictive value of this M2-like TAM-related prognostic model and *VARS1* as a predictive biomarker of anti-PD-1 immunotherapy response in melanoma. In addition, further animal experiments are necessary for exploring the functional role of VARS1 in melanoma, which can help provide more robust clues to guide clinical application.

## Conclusion

Our studies identified a M2-like TAM-related prognostic model for predicting OS and immunotherapy resistance in melanoma and explored the potential predictive value of *VARS1* in melanoma immunotherapy. We hope that our research widens the current understanding of the role of M2-like TAMs in the biology of melanoma and prognosis prediction and that *VARS1* can be a novel predictive biomarker of clinical outcome and immunotherapy response in melanoma.

**Figures and legends**.

## Electronic supplementary material

Below is the link to the electronic supplementary material.

Supplementary Material 1

Supplementary Material 2

## Data Availability

All the data corresponding to the DLBCL series used in this study are available. in GEO (https://www.ncbi.nlm.nih.gov/geo) and TCGA (https://portal.gdc. cancer.gov/), which are public functional genomics data repositories.

## Abbreviations

ARSs: aminoacyl-tRNA synthetases
AUC: area under the curve
CNA: copy number alteration
CTL: cytotoxic T lymphocyte
DEGs: differentially expressed genes
DMEM: Dulbecco’s modified Eagle’s medium
DMPs: differentially methylated probes
FACS: fluorescence-activated cell sorting
FBS: fetal bovine serum
FDR: false discovery rate
FPKM: fragments per kilobase transcript per million fragments
GDC: Genomic Data Commons
GEO: Gene Expression Omnibus
GSEA: gene set enrichment analysis
GSVA: gene set variation analysis
HR: hazard ratio
IL: interleukin
KEGG: Kyoto Encyclopedia of Genes and Genomes
lasso: least absolute shrinkage and selection operator
MFG-E8: milk fat globule-epidermal growth factor VIII
MSigDB: Molecular Signatures Database
OS: overall survival
PBS: phosphate-buffered saline
PMA: phorbol-12-myristate-13-acetate
PPI: protein–protein interaction
qRT-PCR: quantitative real-time PCR
RNA-seq: RNA sequencing
SCNV: somatic copy number variation
siRNA: small interfering RNA
SKCM: skin cutaneous melanoma
SMG: significantly mutated genes
TAMs: tumor-associated macrophages
TCGA: The Cancer Genome Atlas
TMB: tumor mutation burden
TME: tumor microenvironment
TPM: transcripts per million
WGCNA: weighted gene co-expression network analysis
MESO: Mesothelioma
KICH: Kidney Chromophobe
SARC: Sarcoma
LAML: Acute Myeloid Leukemia
CESC: Cervical squamous cell carcinoma and endocervical adenocarcinoma
READ: Rectum adenocarcinoma
