# Supplementary material for: Transcriptome-based network analysis related to M2-like tumor-associated macrophage infiltration identified VARS1 as a potential target for improving melanoma immunotherapy efficacy
Source: J Transl Med. 2022 Oct 27;20:489. doi: 10.1186/s12967-022-03686-z (PMC9615154; doi:10.1186/s12967-022-03686-z)
Supplement: Supplementary file 2 — Supplementary Material 2 [file 12967_2022_3686_MOESM2_ESM.pdf]

Table S1 Univariate and multivariate analyses of clinicopathological characteristics and risk score with overall survival in TCGA SKCM cohort and GEO cohort

	**Univariate analysis**		**Multivariate analysis**	
	HR (95% CI)	P value	HR (95% CI)	P value
**TCGA SKCM (n=467)**				
Gender<br>(Female VS Male)	1.106(0.906-1.349)	0.322		
Clark_level<br>(IV-V VS I-III)	2.086(1.47-2.96)	<0.001	1.294(0.875-1.913)	0.197
Pathologic_stage<br>(III-IV VS I-II)	1.644(1.23-2.198)	<0.001	1.643(1.173-2.302)	0.004
Age<br>(≥ 65 VS < 65)	0.951(0.78-1.158)	0.616		
Radiation_therapy<br>(Yes VS < No)	1.253(0.949-1.653)	0.111		
Breslow_depth<br>(≥ 2.0 VS < 2.0)	0.932(0.749-1.161)	0.531		
Risk Score	2.718(2.205-3.351)	<0.001	2.261(1.735-2.948)	<0.001
**GSE65904 (n=214)**				
Gender<br>(Female VS Male)	1.225(0.974-1.541)	0.082		
Age<br>(≥ 65 VS < 65)	1.152(0.928-1.43)	0.201		
Risk Score	5.123(2.219-11.825)	<0.001	5.123(2.219-11.825)	<0.001

Supplemental Figures and Figure Legends

Supplemental Figure 1

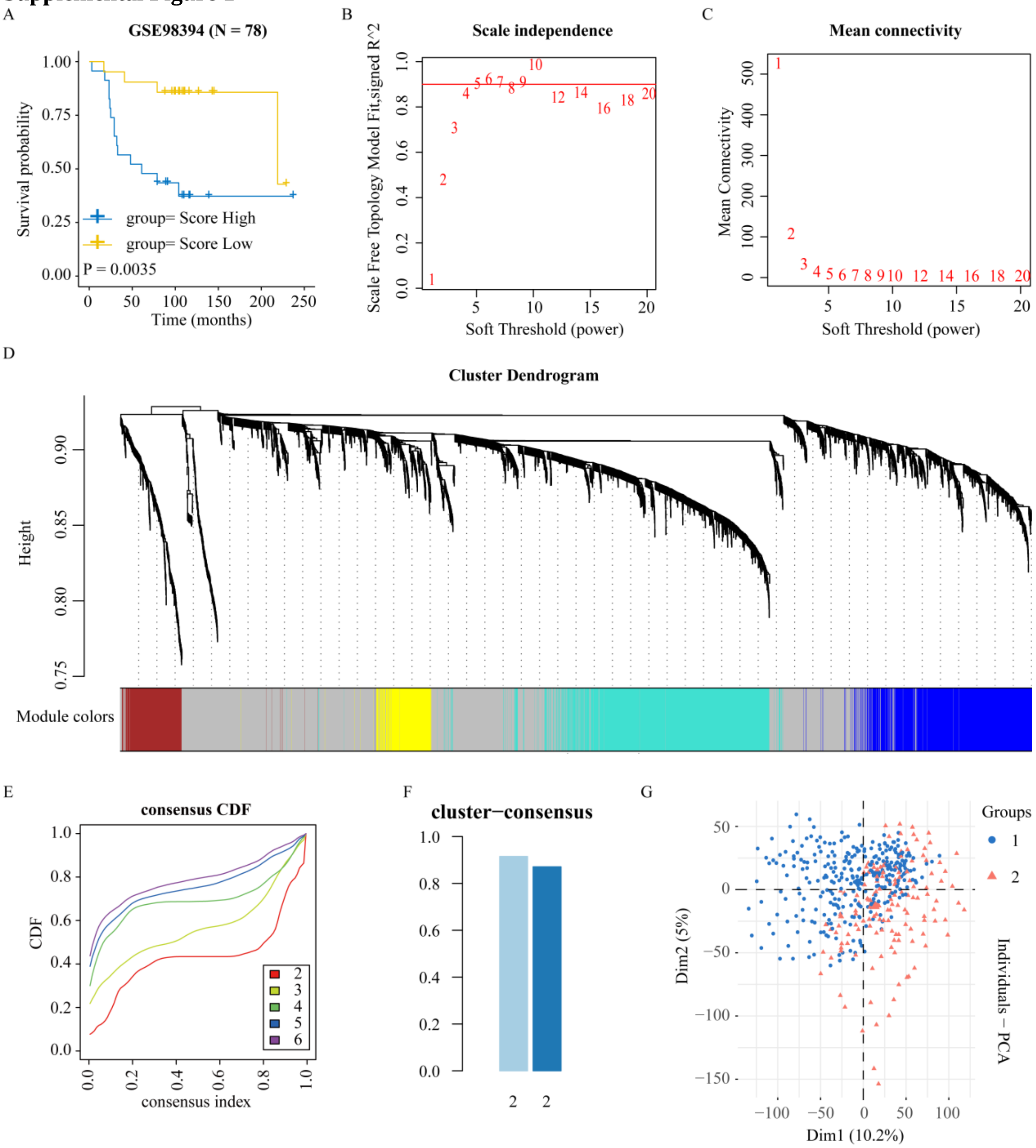

Supplemental Figure 1 Perform WGCNA to identified gene modules and use the consensus clustering to define the stable cluster in TCGA melanoma samples. (A) Kaplan–Meier analysis showing the correlations between M2-like TAMs infiltration and overall survival (OS) in GSE98394 cohorts. Patients were grouped into "high" or "low" groups based on the median CIBER-SORT-based M2 macrophages score. The panel showed that the scale-free fit index (B) and Mean connectivity (C) for different selection of soft-thresholding powers (β). (D) The hierarchical gene dendrogram and module color of TCGA SKCM datasets. (E) Cumulative distribution functions (CDFs) for 2 to 6 consensus clusters. (F) The figure shows the cluster consensus value of 2 cluster. (G) PCA plot showing the distribution of patients in the two clusters.

Supplemental Figure 2

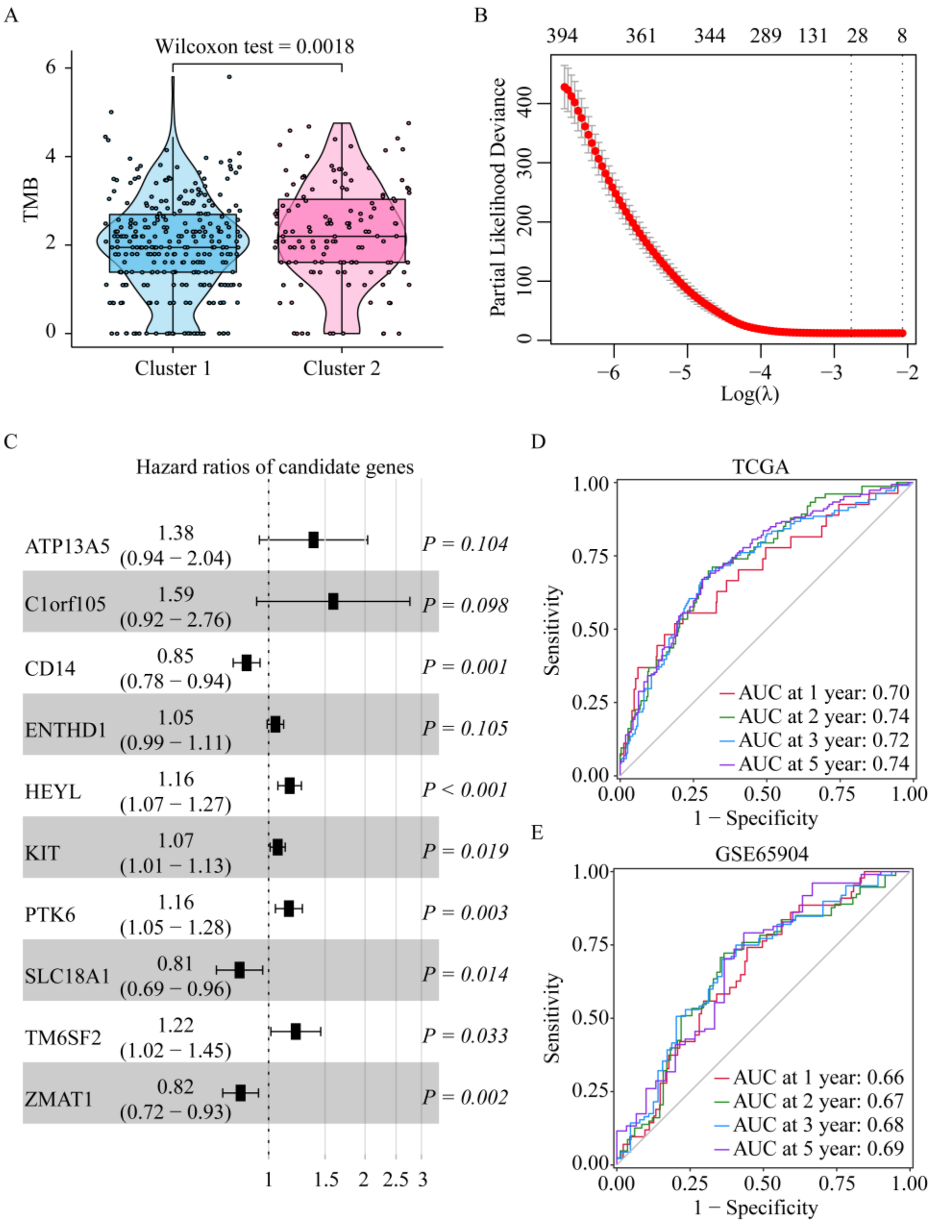

Supplemental Figure 2 TMB analysis of SKCM clusters and construction of Lasso-cox model. (A) The difference of tumor Mutational Burden (TMB) in different clusters. (B) 10-fold cross-validation to get optimal parameters for LASSO models. (C) Forest plot shows the results of multivariable Cox proportional Hazard regression analysis. (D-E) AUC values of the ROC curves for 1-, 2-, 3- and 5-year OS in TCGA and GSE65904.

Supplemental Figure 3

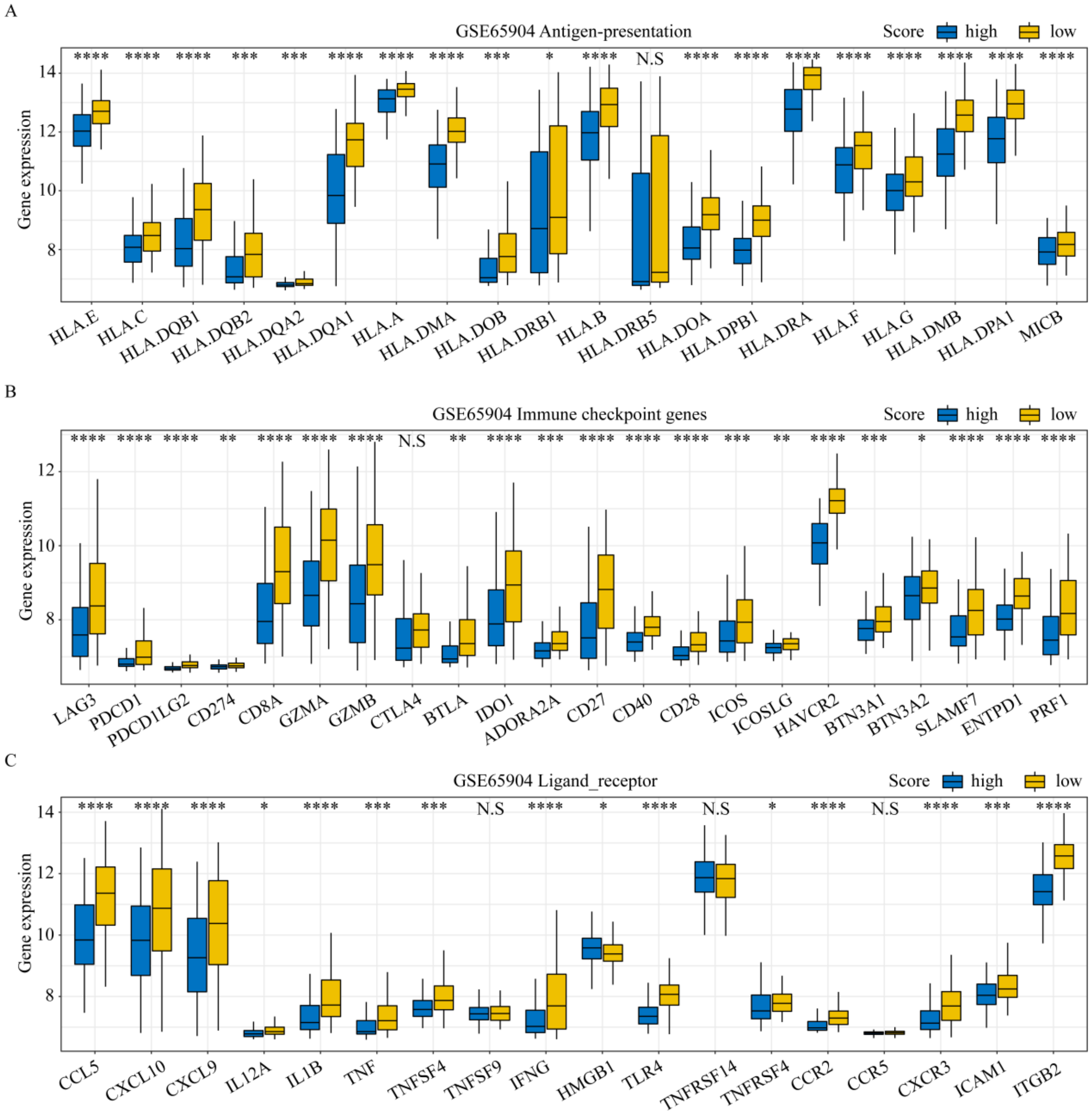

Supplemental Figure 3 Boxplots displayed the differences in the expression of antigen presentation, immune check point genes and several ligand-receptor between GSE65904 high- and low-risk group.

Supplemental Figure 4

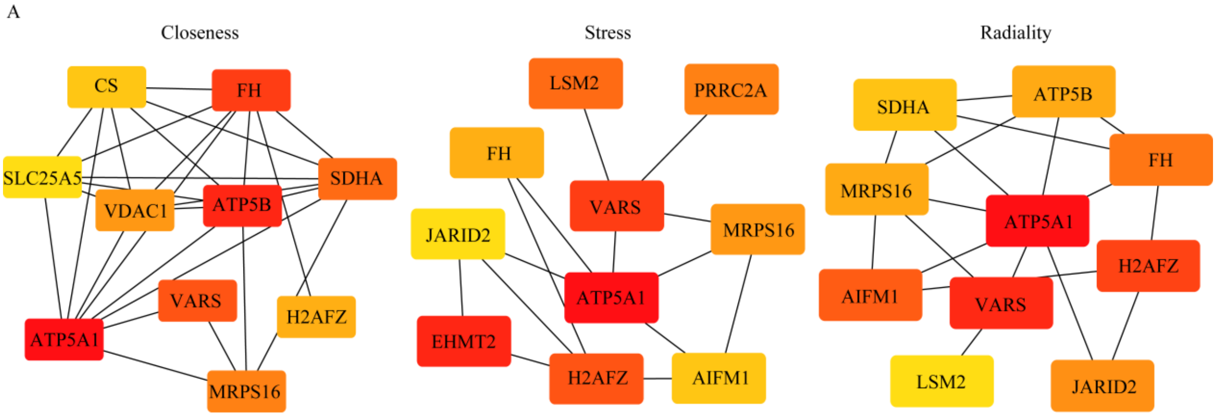

Supplemental Figure 4 The hub genes of M2-like TAMs infiltration correlated modules calculated with three algorithms based on PPI network.

Supplemental Figure 5

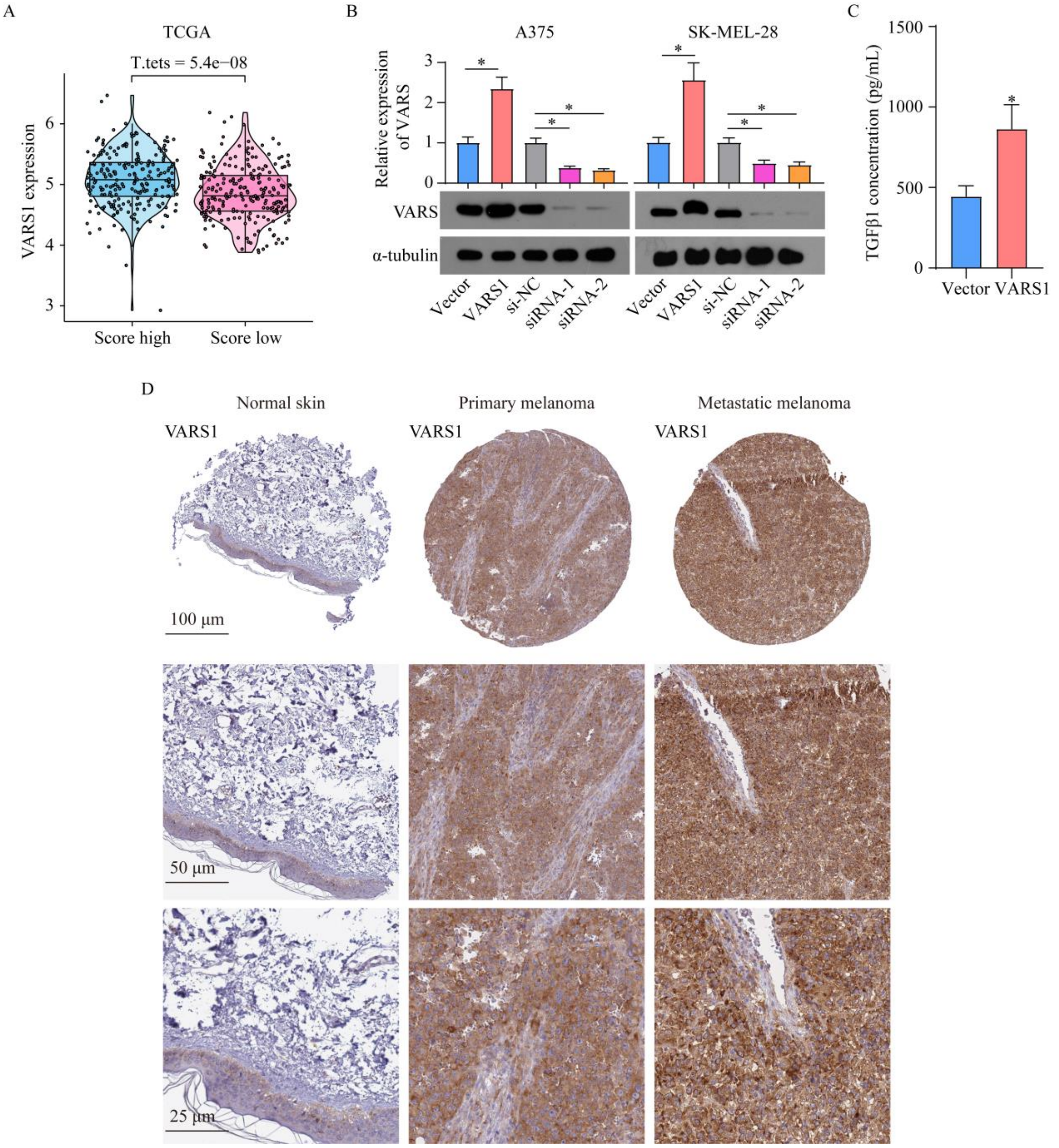

Supplemental Figure 5 The essential role of VARS1 in melanoma progression. (A) Differences in VARS1 expression between high and low risk groups in TCGA. (B) Overexpressing or silencing VARS1 efficiency was verified by Western blot and RT-qPCR analysis. '*' represents p-value < 0.05. (C) The concentration of TGF-β1 in VARS1-overexpressed or Vector A375 cells supernatant was detected by ELISA. '*' represents pvalue < 0.05. (D) Immunohistochemical staining revealed that VARS1 expression in normal skin, primary melanoma and metastatic melanoma from the HPA database.

Supplemental Figure 6

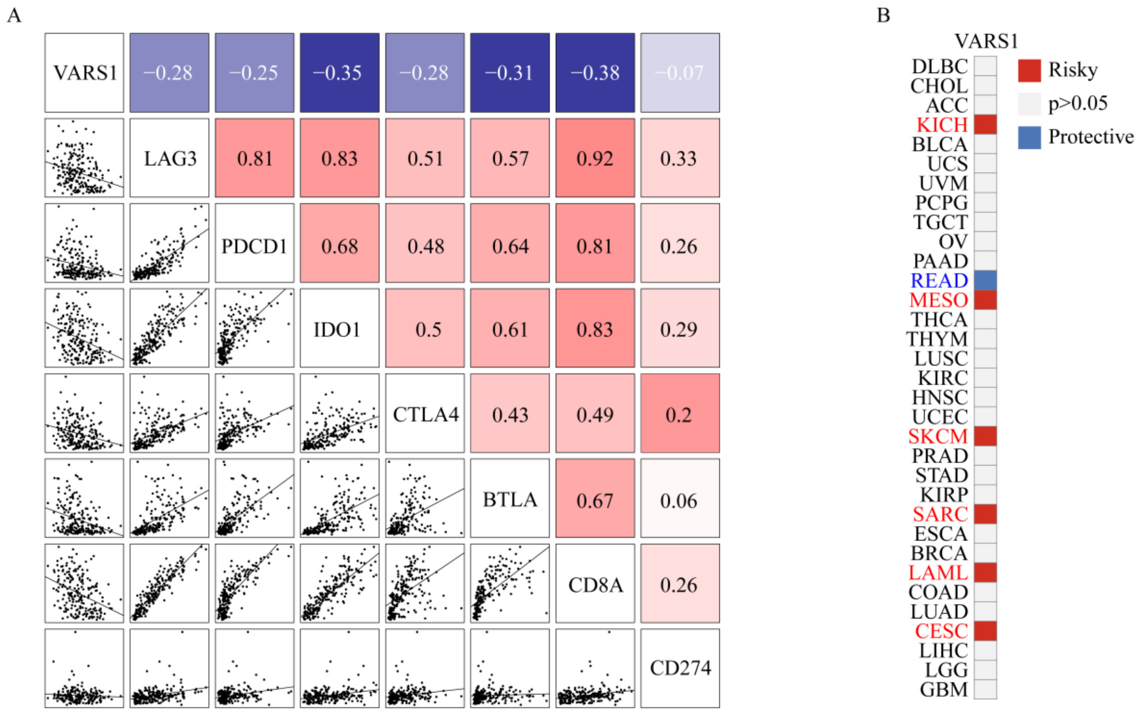

Supplemental Figure 6 The role of VARS1 in cancer prognosis. (A) Correlation between the expression of VARS1 and several known immune checkpoint genes in the GSE65904 cohort. The correlation coefficients were calculated by the Pearson correlation test. (B) Survival risk relevance of VARS1 across TCGA all cancer types. Red indicates that high VARS1 expression predicts poor prognosis, while blue indicates the opposite. Only p values < 0.05 are presented.
